# Increasing HbA1c is associated with reduced CD8^+^ T cell functionality in response to influenza virus in a TCR-dependent manner in individuals with diabetes mellitus

**DOI:** 10.1007/s00018-023-05010-4

**Published:** 2024-01-12

**Authors:** Katina D. Hulme, Zhen Wei Marcus Tong, Louise C. Rowntree, Carolien E. van de Sandt, Katharina Ronacher, Emma J. Grant, Emily S. Dorey, Linda A. Gallo, Stephanie Gras, Katherine Kedzierska, Helen L. Barrett, Kirsty R. Short

**Affiliations:** 1 School of Chemistry and Molecular Biosciences, The University of Queensland, St Lucia, QLD Australia; 2 Department of Medical Microbiology & Infection Prevention, Amsterdam University Medical Center, University of Amsterdam, Amsterdam, The Netherlands; 3 Department of Microbiology and Immunology, University of Melbourne, at the Peter Doherty Institute for Infection and Immunity, Parkville, VIC Australia; 4 Department of Hematopoiesis, Sanquin Research and Landsteiner Laboratory, Amsterdam UMC, University of Amsterdam, Amsterdam, The Netherlands; 5 Mater Research Institute, Translational Research Institute, The University of Queensland, Brisbane, Australia; 6 Australian Infectious Diseases Research Centre, The University of Queensland, St Lucia, QLD Australia; 7 Department of Biochemistry and Chemistry, La Trobe Institute for Molecular Science, La Trobe University, Bundoora, VIC Australia; 8 Department of Biochemistry and Molecular Biology and Infection and Immunity Program, Biomedicine Discovery Institute, Monash University, Clayton, VIC Australia; 9 School of Biomedical Sciences, The University of Queensland, St Lucia, QLD Australia; 10 School of Health and Behavioural Sciences, University of the Sunshine Coast, Moreton Bay, QLD Australia; 11 Obstetric Medicine, The Royal Hospital for Women, Randwick, NSW Australia; 12 School of Medicine, UNSW, Randwick, NSW Australia

**Keywords:** Adaptive immune response, T cell receptor complex, Hyperglycemia, High glucose

## Abstract

Diabetes mellitus is on the rise globally and is a known susceptibility factor for severe influenza virus infections. However, the mechanisms by which diabetes increases the severity of an influenza virus infection are yet to be fully defined. Diabetes mellitus is hallmarked by high glucose concentrations in the blood. We hypothesized that these high glucose concentrations affect the functionality of CD8^+^ T cells, which play a key role eliminating virus-infected cells and have been shown to decrease influenza disease severity. To study the effect of hyperglycemia on CD8^+^ T cell function, we stimulated peripheral blood mononuclear cells (PBMCs) from donors with and without diabetes with influenza A virus, anti-CD3/anti-CD28-coated beads, PMA and ionomycin (PMA/I), or an influenza viral peptide pool. After stimulation, cells were assessed for functionality [as defined by expression of IFN-γ, TNF-α, macrophage inflammatory protein (MIP)-1β, and lysosomal-associated membrane protein-1 (CD107a)] using flow cytometry. Our results showed that increasing HbA1c correlated with a reduction in TNF-α production by CD8^+^ T cells in response to influenza stimulation in a TCR-specific manner. This was not associated with any changes to CD8^+^ T cell subsets. We conclude that hyperglycemia impairs CD8^+^ T cell function to influenza virus infection, which may be linked with the increased risk of severe influenza in patients with diabetes.

**Supplementary Information:**

The online version contains supplementary material available at 10.1007/s00018-023-05010-4.

## Introduction

Influenza A virus (IAV) presents a continuous threat to human health, killing approximately half a million people annually [1, 2]. Influenza severity largely depends on the immune and health status of the individual [3, 4]. Notably, underlying health conditions, such as diabetes mellitus are associated with severe influenza outcomes [5].

The global prevalence of diabetes has been steadily rising for several decades [6]. The continuous rise is driven primarily by type 2 diabetes, which is associated with urbanization and lifestyle factors [6]. While the underlying cause differs, diabetes subtypes are characterized by chronically high concentrations of blood glucose (hyperglycemia) [7]. Analysis of glycated hemoglobin in blood via a HbA1c test provides an indicator of glycaemic control over the preceding 3-month period [8]. Hyperglycemia especially has been identified as a risk factor for developing non-communicable complications associated with diabetes, including cardiovascular disease, nephropathy, and retinopathy [9]. In addition to its role as a risk factor for non-communicable disease, there is rising evidence that hyperglycemia is associated with increased susceptibility to infectious disease and severe outcomes [5, 10, 11]. Specifically, there is evidence to suggest that hyperglycemia has a profound effect on the cellular adaptive immune response, such as that of CD8^+^ T cells [12–18].

The largest body of work suggests that hyperglycemia has detrimental effects on T cells. Specifically, outside the context of influenza virus infection, it has been suggested that hyperglycemia impairs T cell responses, increases the frequency of senescent cells, and impairs their proliferation [12–14]. In vitro, high glucose concentrations reduce the CD8^+^ T cell production of TNF-α, IFN-γ, and IL-12 [15, 16], and reduce cell viability [17]. Consistent with these experimental data, clinically, CD8^+^ T cells from donors with diabetes have a reduced target cell lysis ability compared to control donors without diabetes following influenza vaccination [18]. Furthermore, genome-wide expression analysis of peripheral blood mononuclear cells (PBMCs) from donors with and without diabetes showed a significant reduction in activity of cytotoxic genes associated with diabetes [19]. While there is a clear relationship between diabetes and altered CD8^+^ T cell function in a non-infectious context, these results cannot be directly extrapolated and applied to infectious pathogens. Furthermore, no study to date has investigated the effect of hyperglycemia on CD8^+^ T cell function in the context of influenza.

Currently, there are two subtypes of IAV that circulate in the community, H1N1 and H3N2, and influenza pandemics often (although not always) are associated with the introduction of a new IAV subtype into the human population [20]. While each virus is antigenically different, CD8^+^ T cells typically recognize conserved epitopes between influenza strains and provide heterosubtypic immunity [21], where exposure to one influenza serotype can afford protection to another [22]. Heterosubtypic immunity plays a crucial role in protection against both seasonal infection, and pandemic scenarios [23, 24]. Thus, any reduction in the function of CD8^+^ T cells in response to high concentrations of glucose could possibly impair this heterosubtypic immunity to influenza virus. Together this suggests that, in the context of IAV infection, hyperglycemia may impair the development of poly-functional heterosubtypic immunity to influenza virus by reducing CD8^+^ T cell response to stimulation. By identifying the relationship between glucose concentrations and anti-viral immunity, this study sheds light on one of the mechanisms contributing to increased risk for severe influenza virus infection in individuals with diabetes.

## Materials and methods

### Patient and control recruitment

A cohort of 88 individuals were recruited for the study. The cohort consisted of 64 individuals with type 1 diabetes, 8 individuals with type 2 diabetes, and 16 healthy individuals without diabetes or other health conditions. Inclusion criteria for the donors with diabetes comprised 18–60 years of age, not pregnant at the time of study, non-smokers of nicotine cigarettes, no known immune disease requiring immuno-suppressants, and a minimum of 2-year duration of diabetes mellitus. Inclusion criteria for the donors without diabetes comprised 18–60 years of age, not pregnant at the time of study, non-smokers of nicotine cigarettes, and no underlying health conditions. Non-fasting blood and clinical data from these participants were collected at the point of recruitment. Informed consent was obtained from all subjects, and the study was approved by Mater Misericordiae Ltd Human Research Ethics Committee (Approval No. HREC/17/MHS/78) and the University of Queensland Humans Research Ethics Committee (Approval No. 2019/HE002522).

### PBMC isolation

10 mL of non-fasting blood was collected from donors in BD Vacutainer EDTA tubes (BD Biosciences). PBMCs were isolated from donors with and without diabetes whole blood by Ficoll-Paque (GE Healthcare, Illinois, USA) gradient centrifugation. Cells were cryopreserved in fetal bovine serum (FBS) (Sigma-Aldrich) containing 10% dimethyl sulfoxide and stored below − 135°C until tested.

### Ex vivo T cell characterization

**Fig. 1:**
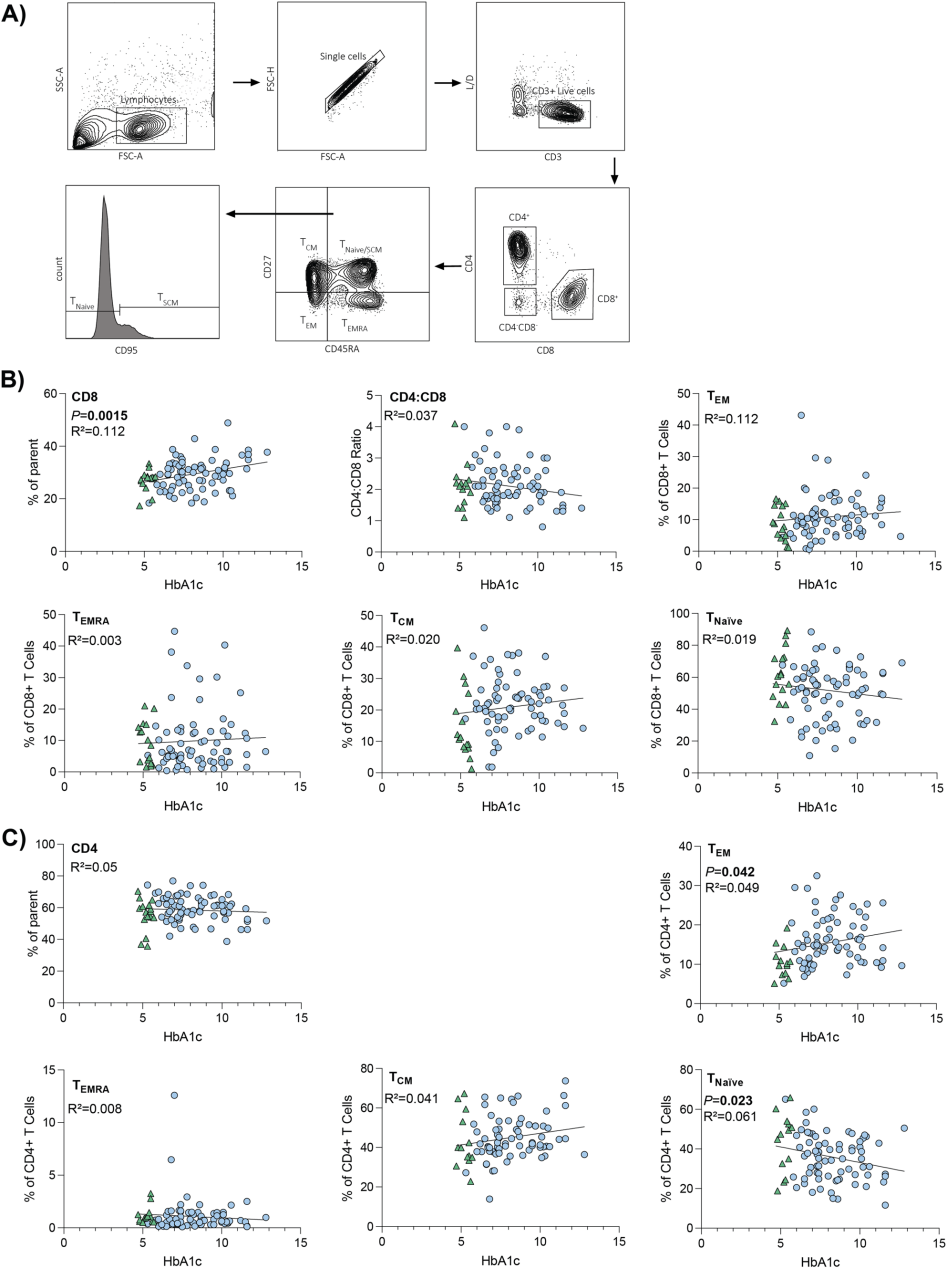
Increasing HbA1c correlated with changes to CD4^+^ T cell subset populations. **A** Gating strategy employed to characterize CD8^+^ and CD4^+^ T cell populations. Fluorochrome-labeled antibodies were used to characterize the T cell populations. PBMCs were stained for lymphocyte and differentiation markers as outlined in the methods. **B** Correlation analysis between % HbA1c and CD8^+^ TCentral Memory (Tcm), TNaïve, TEffector Memory (T_EM_) and T terminally differentiated effector memory (T_ERMA_) subsets, overall CD8^+^ T cell population, and CD4:CD8 ratio. Data points represent individual donors (*n* = 88). Statistical significance was determined using simple linear regression, with significant *P* values displayed. **C** Correlation analysis between % HbA1c and CD4^+^ TCentral Memory, TNaïve, TEffector Memory and T terminally differentiated effector memory (TERMA), and overall CD4^+^ T cell population. Data points represent individual donors (*n* = 88). Statistical significance was determined using simple linear regression, with significant *P* values displayed.** B**, **C** Donors without diabetes are represented by green triangles. Donors with diabetes are represented by blue circles

Characterization of the T cell populations was performed as previously described [25]. In short, PBMCs were washed and stained for lymphocyte (anti-human CD3-BV480, anti-human CD4-BV650, and anti-human CD8-PerCPCy5.5), and differentiation (anti-human CD27-APC, anti-human CD45RA-FITC, and anti-human CD95-BV421). Cells were subsequently washed and fixed using a cytofix kit (BD Biosciences). All samples were analyzed by flow cytometry on LSRFortessa (BD Biosciences). The gating for T cell characterization is outlined in Fig. 1a.

### T cell stimulation and intracellular cytokine staining (ICS)

**Fig. 2:**
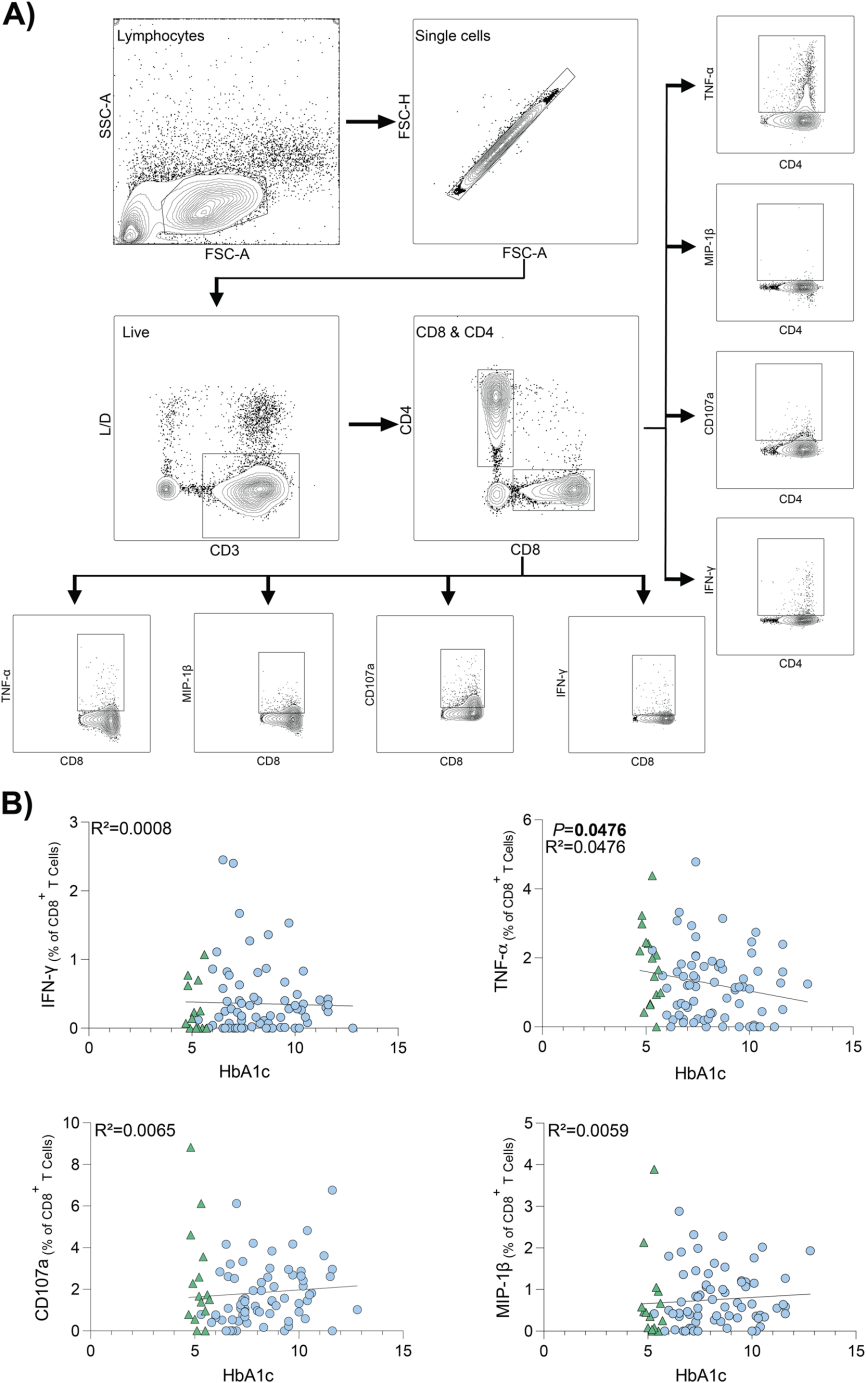
Percentage of CD8^+^ T cells expressing TNF-α following IAV infection inversely correlates to HbA1c. **A** Gating strategy employed to investigate CD8^+^ and CD4^+^ T cell response to stimulation. Fluorochrome-labeled antibodies were used to identify the CD4^+^ and CD8^+^ T cell populations expressing IFN-γ, TNF-α, MIP1-β and CD107a. **B** Correlation analysis between % HbA1c and frequency of cells expressing IFN-γ, TNF-α, CD107a and MIP-1β, with background staining subtracted. CD8^+^ T cells were infected with IAV (HKx31, H3N2) for 18 h. Results are relative to negative control condition (unstimulated cells). Data points represent individual donors (*n* = 88). Statistical significance was determined using simple linear regression, with significant *P* values displayed. **A**, **B** Donors without diabetes are represented by green triangles. Donors with diabetes are represented by blue circles

PBMCs from donors with and without diabetes were stimulated with either (i) A/H3N2 (HKx31) at a MOI of 10, (ii) PMA (1 µg/mL) and ionomycin (1 µg/mL), (iii) influenza virus peptide pool (4 µg/mL) (Table 1) (AnaSpec), (iv) CD3/CD28 magnetic beads (Thermo Fisher), or (v) left unstimulated for 18 h in the presence of anti-human CD107a-FITC (Biolegend), GolgiStop (BD Biosciences) and GoliPlug (BD Biosciences). Cells were then washed, fixed, and stained with anti-human CD8-PerCPCy5.5 (BD Biosciences) anti-human CD4-PE (BD Biosciences), CD3-PE-Cy7 (BD Biosciences), and NIR live/dead (Invitrogen). Following surface staining, cells were washed and permeabilized using the Cytofix/Cytoperm Fixation/Permeabilization kit (BD Biosciences) and stained with anti-human MIP-1β-APC (BD Biosciences), anti-human IFN-γ-V450 (BD Biosciences) and anti-human TNF-α-AF700 (BD Biosciences). Cells were then analyzed by flow cytometry on LSRFortessa (BD Biosciences) at the flow cytometry facility at the Translational Research Institute (TRI) (Brisbane, Australia). To assess the functionality of T cells, the number of MIP1-β^+^, IFN-γ^+^, TNF-α^+^, and CD107a^+^ cells was assessed using the gating strategy outlined in Fig. 2a.

**Table 1:** Influenza viral peptides included in the peptide pool, together covering 97% of the Caucasian population [26, 27]

HLA Allele	Peptide Sequence	Virus Segment
A1	VSDGGPNLY	Influenza A—PB1
A1	CTELKLSDY	Influenza A—NP
A2	GILGFVFTL	Influenza A—M
A2	FMYSDFHFI	Influenza A—PB2
A68	KTGGPIYKR	Influenza A—NP
A3	RVLSFIKGTK	Influenza A—NP
A3	ILRGSVAHK	Influenza A—NP
A3, A11, A60B1	SIIPSGPLK	Influenza A—M
B7	LPFDKTTVM	Influenza A—NP
B8	ELRSRYWAI	Influenza A—NP
B27	SRYWAIRTR	Influenza A—NP
B27	ASCMGLIY	Influenza A—M

### Statistical analysis

Statistical analyses were performed using Graph Pad Prism software (version 9.3.1) for Windows (GraphPad Software, La Jolla, CA, USA). Statistical significance of categorical patient data was determined using a Fisher’s exact test. The normality of numerical patient data was determined to be not normally distributed, and subsequently Mann–Whitney test was used to determine statistical significance. Simple linear regression was used to determine the relationship between one independent variable and one dependent variable. Multiple linear regression was used to determine the relationship between multiple independent variables (including HbA1c, age, sex, BMI, and ethnicity) and one dependent variable. Independent variables were selected based on prior studies implicating their role in diabetes.

## Results

### Study participant characteristics

The clinical characteristics of donors are summarized in Table 2. Age, sex, and ethnicity were comparable between groups. As expected, both BMI and HbA1c were significantly higher in the cohort of donors with diabetes compared to donors without diabetes.

**Table 2:** Clinical characteristics of donor cohort

	Donors without diabetes (*n* = 16)	Donors with diabetes (*n* = 72)	*P*-value
Age, years	28.1 ± 5.4	30.6 ± 11.6	0.874
Female/male (% female)	11/5 (68.8%)	44/28 (60.3%)	0.776
BMI (kg/m^2^)	23 ± 4.6	27 ± 5.4	0.006 (*)
Ethnicity, % caucasian	68.8%	83.6%	0.157
HbA1c (%)	5.2 ± 0.3	8.4 ± 1.7	< 0.0001 (*)
T1DM/T2DM (% T1DM)	N/A	66/6 (90.4%)	N/A
Insulin treatment	N/A	66/6 (90.4%)	NA
Duration of diabetes (years)	N/A	14.8 ± 7.2	N/A
Non-steroidal anti-inflammatory drug (NSAID) use (% use)	0/16 (0%)	4/68 (5.5%)	> 0.999

Values are mean ± SD unless otherwise indicated

T1DM, Type 1 Diabetes mellitus; T2DM, Type 2 Diabetes mellitus; BMI, Body mass index

Significant *P* values indicated by an asterisk (*)

### Increasing HbA1c associated with changes in ex vivo T cell populations

To assess if hyperglycemia was associated with changes to both CD4^+^ and CD8^+^ T cell populations, PBMCs were stained for lymphocyte and phenotypic markers.

Increasing HbA1c correlated with an increased frequency of CD8^+^ T cells (defined as frequency of parent population). However, this increase was not enough to affect the overall CD4:CD8 ratio (Fig. 1b). This increase in proportion of CD8^+^ T cell population is characteristic of type 1 diabetes [28]. There was no correlation between HbA1c and distribution of central memory, naïve, effector memory, and terminally differentiated effector memory subsets of the CD8^+^ T cell population (Fig. 1b). These data suggest that the hyperglycemia increased the overall proportion of CD8^+^ T cells but not their phenotypes.

In contrast, increasing HbA1c was associated with an increasing proportion of effector memory and decreasing proportion of naïve CD4^+^ T cell populations (Fig. 1c). Alterations CD4^+^ T cell populations such as these are commonly observed in patients with diabetes [29, 30].

Together these data suggest that HbA1c is associated with changes to T cell populations that are typically observed in individuals with diabetes.

### Expression of TNF-α from CD8^+^ T cells following IAV infection correlated to HbA1c

To assess the effect of HbA1c on influenza virus-specific CD8^+^ T cell function and recall ability, PBMCs were infected with IAV for 18 h. Influenza virus-induced CD8^+^ T cell functionality was defined by expression of IFN-γ, TNF-α, MIP1-β, and CD107a (Fig. 2a). Using simple linear regression analysis, there was no correlation between HbA1c and CD8^+^ T cell expression of IFN-γ, MIP1-β, or CD107a (Fig. 2b). However, increasing HbA1c correlated with reduced proportion of TNF-α producing CD8^+^ T cells (Fig. 2b). In addition to the assessment of single activation markers, polyfunctionality of CD8^+^ T cells was also assessed. However, there was no significant effect of HbA1c (data not shown).

### Decreased TNF-α expression is specific to the activation of the TCR complex

Having established that increased HbA1c is associated with decreased TNF-α expression following influenza virus infection, we next sought to replicate these findings under more controlled conditions and to determine if this effect was specific to influenza virus stimulation.

The magnitude of peptide-specific cells is known to vary greatly between HLA-matched individuals and may be influenced by infection or vaccination history, and other HLA molecules present [31–33]. As such, it would be difficult to determine whether hyperglycemia has an effect on peptide-specific CD8^+^ T cells event in individuals that are HLA-matched for that peptide's restricting HLA molecule.

Therefore, rather than stimulation with HLA-matched peptides, we chose to expose the PBMCs to either an influenza viral peptide pool containing 12 influenza viral peptides spanning 10 HLA subtypes (Table 1) or beads coated with anti-CD3/anti-CD28. Both the peptide pool and the beads stimulate CD8^+^ T cells via the TCR complex. Stimulation with PMA/I is known to bypass the required binding to the TCR complex on the cell surface [34]. Instead, ionomycin (a calcium ionophore) facilitates the transport of calcium across the plasma membrane and directly increases the intracellular Ca^2+^, while PMA activates protein kinase C, leading to the activation of MAPK pathways.

**Fig. 3:**
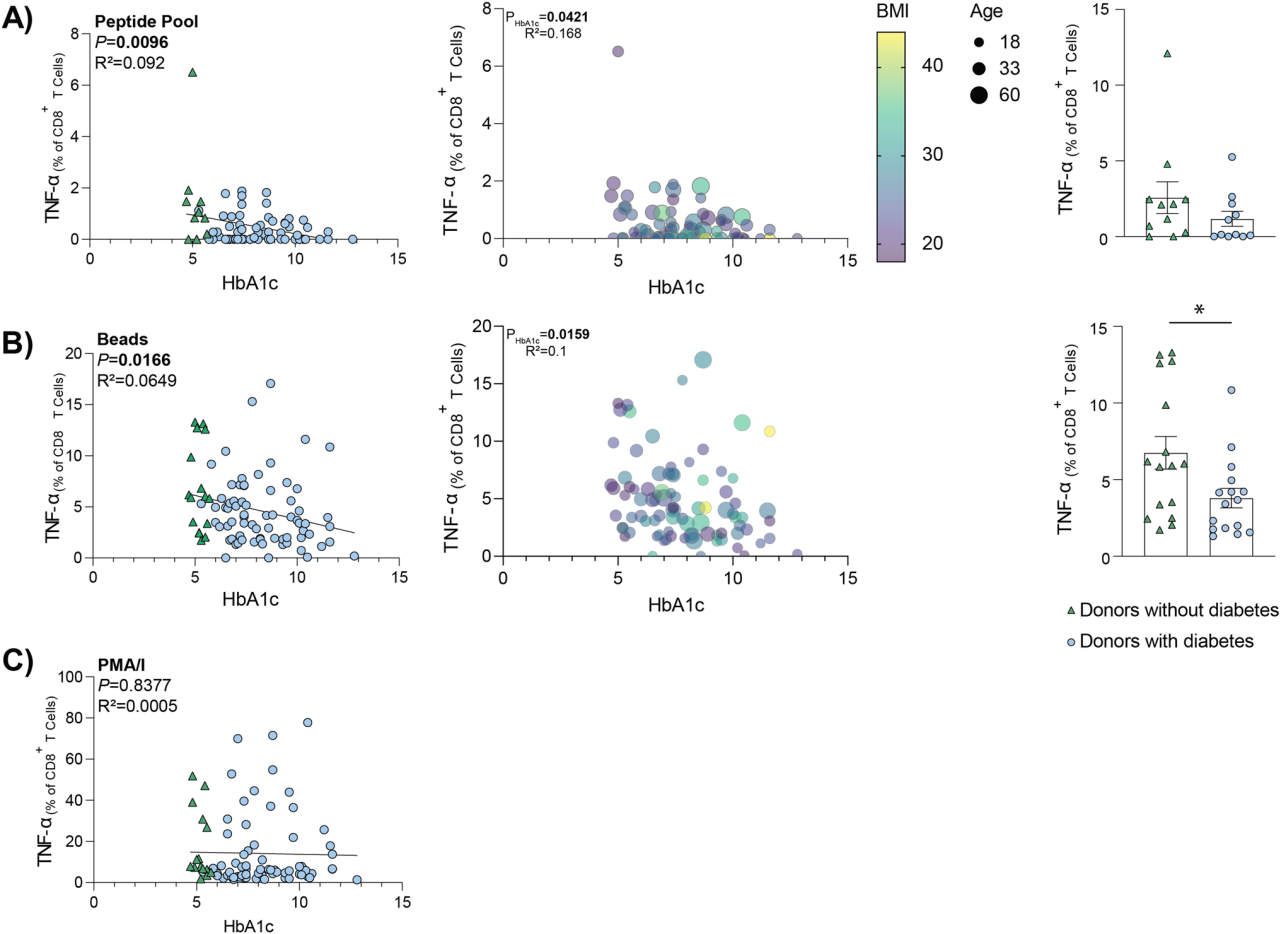
The association between HbA1c and TNF-α expression was observed following stimulation in a TCR-specific manner. CD8^+^ T cells were stimulated for 18 h using either, influenza virus peptide pool (**A**), anti-CD3/anti-CD28 coated beads (**B**), or PMA/I (**C**). Results are presented as the frequency of CD8^+^ T cells expressing TNF-α, with background staining subtracted. Data points represent individual donors (left & middle: Beads and PMA/I *n* = 88, peptide pool *n* = 73 [Caucasian donors]) (right: age- and sex-matched donors [*n* = 11/group (**A**), *n* = 16/group (**B**)]. Statistical significance was determined using simple linear regression (left), multiple variable regression analysis, where input variables were age, sex, BMI, HbA1c and ethnicity (middle), or a Mann–Whitney test *P < 0.05 (right). **A**–**C** Donors without diabetes are represented by green triangles. Donors with diabetes are represented by blue circles

Successful activation by a peptide pool stimulation relies on both successful antigen presentation of the peptide, and on donors having a requisite HLA subtype that correspond to a peptide present in the pool (Table 1). The peptide pool used in this study has a 97% coverage of Caucasian population [27]. Therefore, only Caucasian donors were included for peptide pool analysis. When cells were stimulated with the influenza virus peptide pool, there was an inverse correlation between HbA1c and TNF-α expression by CD8^+^ T cells (Fig. 3a).

While IAV infection, and stimulation with a peptide pool relies on successful antigen presentation by cells, anti-CD3/anti-CD28-coated beads partially mimic the binding of antigen presenting cells to T cells and thus induces TCR stimulation. When cells were stimulated with anti-CD3 and anti-CD8 coated beads, increasing HbA1c correlated with significantly reduced TNF-α expression from responding cells (Fig. 3b). This significant association remains when donors with type 2 diabetes are removed from the analysis (data not shown). Importantly, as this stimulation does not require antigen presentation by target cells, the reduced response can solely be attributed to the effector cells.

In contrast to both the peptide pool and the bead stimulation, when cells were stimulated with PMA/I for 18 h, there was no correlation between the HbA1c and the resulting TNF-α expression (Fig. 3c), indicating that HbA1c specifically affects TCR-induced activation.

Further analysis of the peptide pool stimulation determined that this reduced percentage of TNF-α expressing cells was not due to select confounding factors (specifically, age, sex, or BMI) (Fig. 3a). The response from age- and sex-matched donors was also compared (Fig. 3a). While there was a trend toward decreased response in donors with diabetes, these results did not reach significance, potentially due to the low numbers (11 donors per group).

Similar to the peptide pool stimulations, further analysis of the anti-CD3/anti-CD28-coated beads stimulation also determined that this reduced TNF-α expression was not due to confounding factors (specifically, age, sex, BMI, or ethnicity) (Fig. 3b). When donors were matched for age and sex, the HbA1c-dependent decrease in TNF-α expression following stimulation with beads remained significantly different between the groups (Fig. 3b). When assessing poly-functionality, multiple TNF-α expressing CD8^+^ T cell populations were affected by increasing levels of HbA1c (Supplementary Fig. S1a-d).

Reduced TNF-α expression was also observed in CD4^+^ T cells and significantly associated with both HbA1c and ethnicity of the donor (Supplementary Fig. S2a). When ethnicity was controlled for (i.e., only assessing Caucasian donors), the relationship between increasing HbA1c and reducing TNF-α expression remained (Supplementary Fig. S2b).

Taken together, as both peptide pool and anti-CD3/anti-CD28-coated bead responses were affected, these results suggest that increasing glucose concentrations have a detrimental effect on both CD8^+^ CD4^+^ T cell function upstream of where PMA and ionomycin act, i.e., the initial steps of the TCR signaling cascade.

## Discussion

Hyperglycemia plays an important role in the susceptibility of patients with diabetes to severe influenza [5]. We have previously demonstrated in vitro that high glucose conditions prior to IAV infection increased virus-induced barrier damage [35]. However, it is important to note that our previous work demonstrated the effect of hyperglycemia with no previous exposure to IAV and focused on the innate immune response, rather than adaptive. Most individuals have been exposed to at least one influenza virus by the age of six, and thus will possess a level of adaptive immunity to IAV [36]. Furthermore, it is the adaptive immune response, and more specifically the CD8^+^ T cell response, that offers protection in the form of heterosubtypic immunity, to not only seasonally circulating IAV strains, but also to novel IAV strains with pandemic potential [22, 24, 37]. Given the critical role of CD8^+^ T cells in immune protection against severe influenza, it is imperative to investigate if this protection is altered in individuals with hyperglycemia.

Hyperglycemia has previously been implicated in the redistribution of lymphocyte subsets [38]. Analysis of whole blood lymphocyte count demonstrated a reduction of overall circulating lymphocyte populations in response to short term (2 h) hyperglycemia. This reduction included B cells, and CD4^+^ and CD8^+^ T cells, but subsets were not distinguished [38]. While the results of our study do not report an effect of hyperglycemia on the percentage of CD8^+^ T cell phenotypes (such as naïve central memory, effector memory, and terminally differentiated effector memory), we did observe an increase in the overall proportion of CD8^+^ T cells, consistent with previous findings [28]. Similarly, we report a redistribution of CD4^+^ T cell phenotypes. However, it is important to note that we did not investigate the abundance of influenza-specific CD8^+^ memory T cells, which is reduced in a similar metabolic condition often associated with type 2 diabetes, obesity [39, 40].

Outside the context of influenza, in a non-infectious setting, hyperglycemia causes a five-fold decrease in proliferation of T cells in response to stimulation [12], and has been shown to have a detrimental impact on the survival and function of memory CD8^+^ T cells [15]. As our results demonstrate a reduced ability to respond to TCR stimulation, it is likely that this would affect the downstream proliferation of antigen-specific CD8^+^ memory T cells. However, to confirm this further in vitro proliferation experiments from these donors would be required. Further in vitro experiments may also shed light on whether hyperglycemia has a direct effect on CD8^+^ T cells, or indirectly mediated by other cell populations.

Of the measured markers of CD8^+^ T cell functionality in our cohort, the one significantly affected was TNF-α. TNF-α directly inhibits viral replication [41] and acts as an effector molecule in CD8^+^ T cell-mediated cell lysis [42]. Therefore, any loss of the TNF-α response to influenza virus infection may be detrimental to the individual. Indeed, following influenza virus infection, TNF-α-deficient mice have a dysregulated inflammatory response and significantly increased lung immunopathology [43].

Our results demonstrate that hyperglycemia affects TCR-specific stimulation which hampers CD8^+^ T cell function by reducing the expression of TNF-α. However, hyperglycemia does not prevent their intrinsic ability to function, as demonstrated by the fact that PMA/I stimulation, which circumvents TCR stimulation, did not affect TNF-α expression. Indeed, our results are consistent with studies outside of the context of hyperglycemia, where oxidative stress has been implicated in T cell hypo-responsiveness [44–47]. Therefore, further investigation into hyperglycemia-induced oxidative stress in the context of T cell dysfunction is warranted. This research may hold clues for potential molecular pathways which could be targeted by medication to improve CD8^+^ T cell functionality in a hyperglycemic environment as observed in patients with diabetes mellitus. There are several limitations associated with this study. Firstly, the influenza vaccination status of the control cohort was not recorded. Despite this, it is well established that individuals are no longer immunologically naïve to influenza A viruses by the age of six, and thus possess some level of adaptive immune response to influenza [36]. While the data suggests a role of hyperglycemia in reducing CD8^+^ T cell functionality, the possibility of other confounding factors not measured (e.g., cholesterol and triglyceride levels) need to be considered. For example, dyslipidemia is known to have a profound effect on the immune system, and specifically, can impact the activation and differentiation of T cells [48]. Secondly, we were unable to assess the effect of exogenous insulin as all donors in this study with type 1 diabetes (> 90% of our donors with diabetes) were receiving insulin treatment. While the present study was unable to assess the effect of insulin, Kavazović and colleagues recently demonstrated both in vitro and in vivo that hyperglycemia rather than hyperinsulinemia mediates memory CD8^+^ T cell dysfunction in a mouse model [15].

Originally, we set out to determine the effect of hyperglycemia on influenza specifically, however our conclusions are more far reaching. The results described herein are not limited to influenza virus-specific T cells, but instead to the function, or perhaps the expression, of the TCR complex. Thus, our results are potentially of importance in the context of other pathogens in which diabetes is a risk factor for severe infections, such as *Mycobacterium tuberculosis* (Mtb) and SARS-CoV-2. Our results are consistent with a previous study showing that Mtb antigen-specific CD8^+^ T cells from donors with type 2 diabetes have significantly diminished expression of cytotoxic marker compared to donors without diabetes [49]. In the context of SARS-CoV-2 infection, there are indications that both increased senescence of CD4^+^ and CD8^+^ T cells, as well as the overall reduced proportions of these cell types in patients with diabetes may have contributed to the observed increase in severe COVID-19 [10, 50]. There is also evidence that poorly controlled HbA1c (HbA1c 1-year mean > 7%) following SARS-CoV-2 vaccination in a cohort of patients with type 2 diabetes is associated with a reduced cytokine response from CD4^+^ T cells and increased incidence of breakthrough infections [51]. However, much like influenza, the mechanism underlying this impaired cellular function remains undefined. Therefore, the results of this study may shed some light on the mechanism at play in severe COVID-19 in patients with diabetes.

### Supplementary Information

Below is the link to the electronic supplementary material.

Supplementary file 1 (DOCX 526 KB)

Supplementary file 2 (PDF 640 KB)

## Data Availability

The data sets generated during the current study are not publicly available due to nature of the ethical approvals. If you have a reasonable request, please contact the corresponding author.
