# Supplementary material for: Increasing HbA1c is associated with reduced CD8+ T cell functionality in response to influenza virus in a TCR-dependent manner in individuals with diabetes mellitus
Source: Cell Mol Life Sci. 2024 Jan 12;81(1):35. doi: 10.1007/s00018-023-05010-4 (PMC10786977; doi:10.1007/s00018-023-05010-4)
Supplement: Supplementary file 2 — (PDF 640 KB) [file 18_2023_5010_MOESM2_ESM.pdf]

## Increasing HbA1c is associated with reduced CD8+ T cell functionality in response to influenza virus in a TCR-dependent manner in individuals with diabetes mellitus

Katina D. Hulme[1,2], Zhen Wei Marcus Tong[1], Louise C. Rowntree[3], Carolien E. van de Sandt[3,4], Katharina Ronacher[5,6], Emma J. Grant[7,8], Emily S. Dorey[9], Linda A. Gallo[10,11], Stephanie Gras[7,8], Katherine Kedzierska[3], Helen L. Barrett[9,12,13] & Kirsty R. Short[1,6]

## Supplementary Material

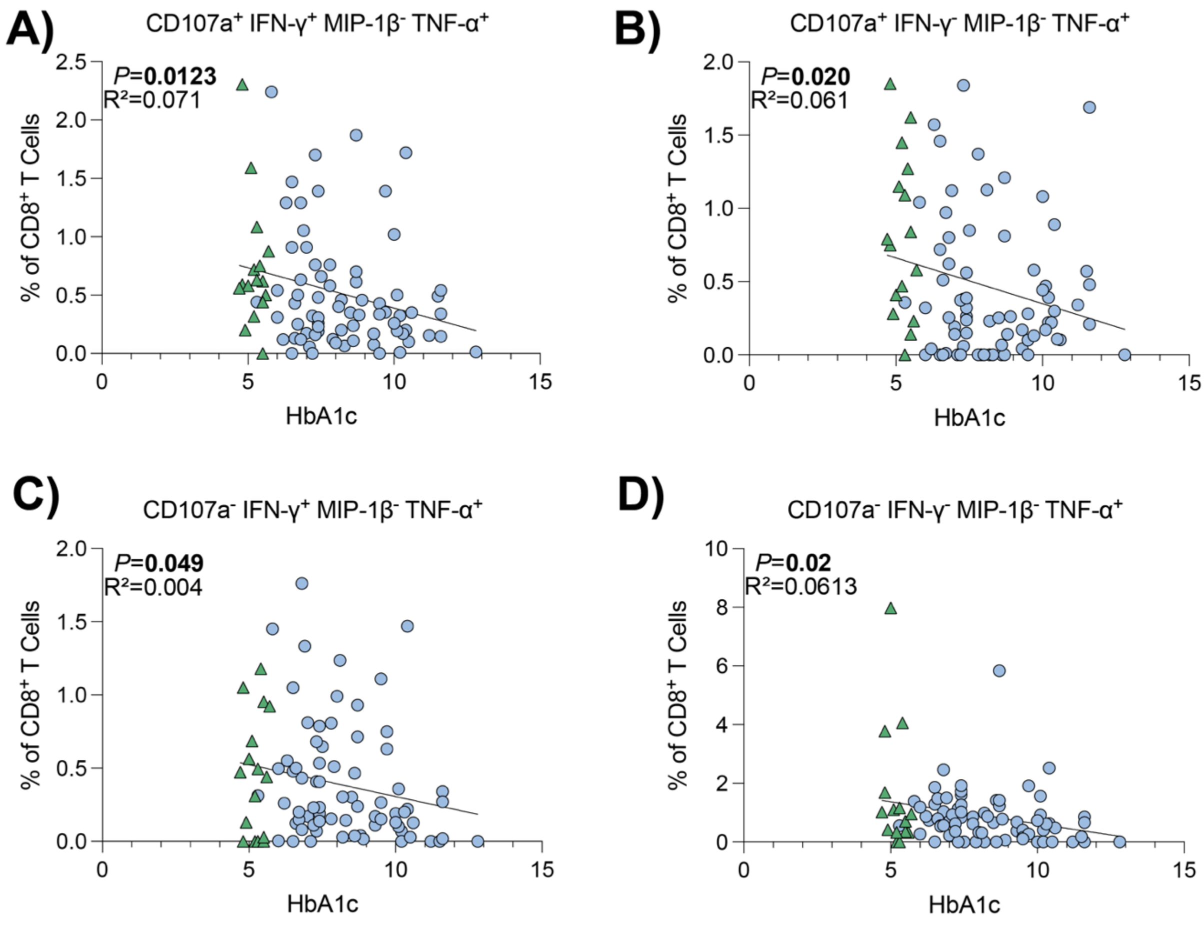


**Supplementary Figure 1: TNF-α expression was affected in multiple polyfunctional CD8+ T cell populations.**

CD8+ T cells were stimulated for 18 hours using anti-CD3/anti-CD28 coated beads. Results are presented as the frequency of CD8+ T cells expressing the select markers, with background staining subtracted. Data points represent individual donors (n=88). Statistical significance was determined using simple linear regression, with significant P values displayed. **A-D)** Donors without diabetes are represented by green triangles. Donors with diabetes are represented by blue circles.

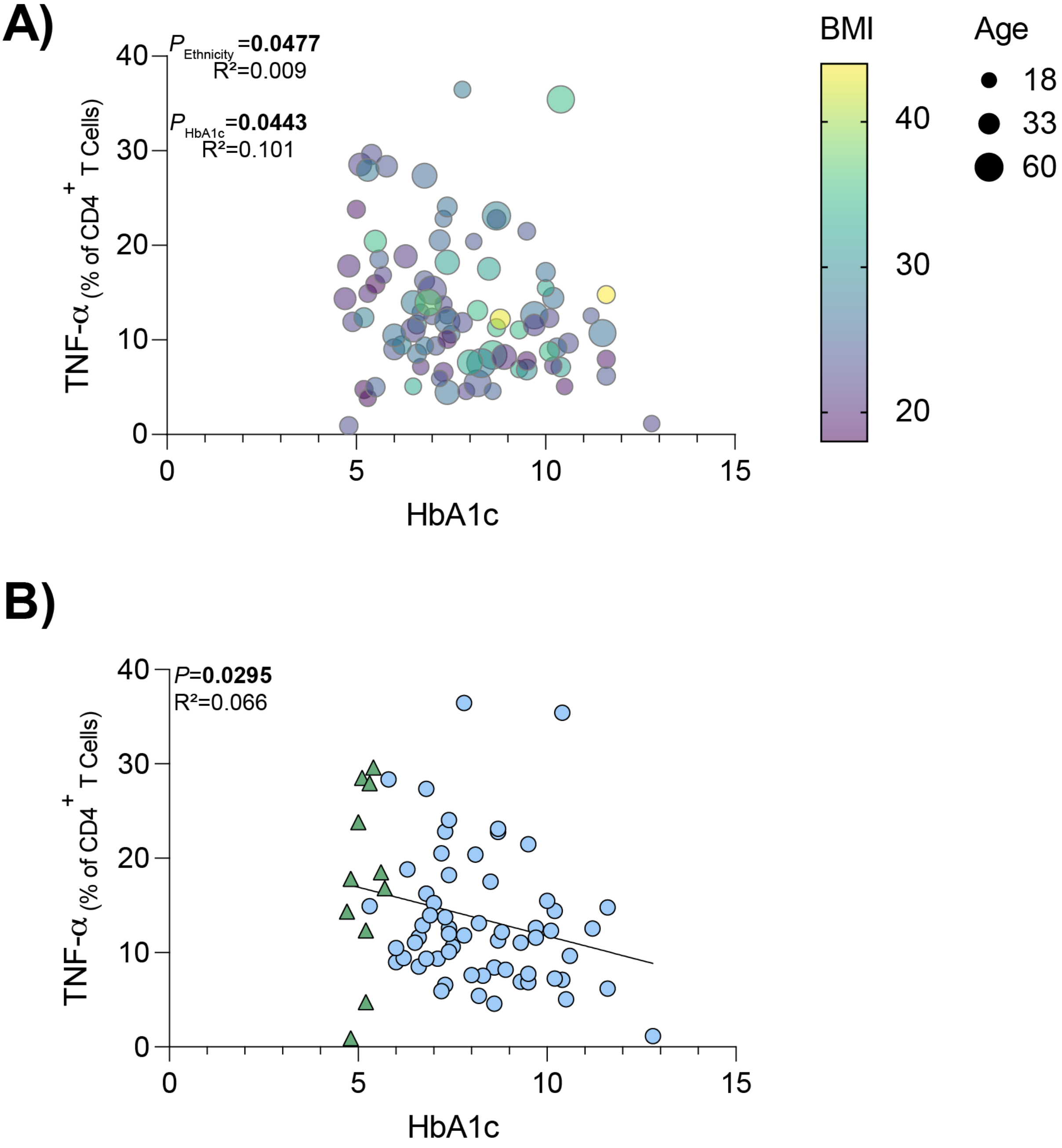


**Supplementary Figure 2: Reduced TNF-α expression associated with increasing HbA1c was also observed in $CD4^+$ T cells.**

$CD4^+$ T cells were stimulated for 18 hours using anti-CD3/anti-CD28 coated beads. Results are presented as the frequency of $CD8^+$ T cells expressing TNF-α, with background staining subtracted. **A)** Data points represent individual donors (n=88). Statistical significance was determined using multiple variable regression analysis, where input variables were age, sex, BMI, HbA1c and ethnicity, with significant *P* values displayed. **B)** Relationship between HbA1c and TNF-α produced by $CD4^+$ T cells in Caucasian donors. Each data point represents an individual donor (n=73). Statistical significance was determined using simple linear regression. Donors without diabetes are represented by green triangles. Donors with diabetes are represented by blue circles.
